# Treatment failure, death, and predictors among PLWHIV on second-line antiretroviral therapy in Dessie Comprehensive Specialized Hospital, northeast Ethiopia: A retrospective cohort study

**DOI:** 10.1371/journal.pone.0269235

**Published:** 2022-06-01

**Authors:** Shambel Wedajo, Getu Degu, Amare Deribew, Fentie Ambaw

**Affiliations:** 1 School of Public Health, CMHS, Wollo University, Dessie, Ethiopia; 2 School of Public Health, CMHS, Bahir Dar University, Bahir Dar, Ethiopia; 3 Country Director, Nutrition International (NI) in Ethiopia, Addis Ababa, Ethiopia; University of Sassari, ITALY

## Abstract

**Background:**

The proportion of HIV patients on second-line antiretroviral therapy is becoming a growing public health concern, especially in a low-income country setting. However, unlike first-line therapy, to date, very little is known about the outcomes of second-line therapy in the Ethiopia context. Thus, this study was conducted to determine the rate of treatment failure, death, and their predictors among HIV patients receiving second-line therapy.

**Methods:**

A retrospective cohort study was conducted on 642 people living with HIV in Dessie Comprehensive Specialized Hospital from October 2016 to November 2019. Poisson and competitive risk survival models were computed to explore predictors of treatment failure and death, respectively.

**Results:**

During follow-up period, 39 (6.87%, 95% CI: 5–9.2%) of 568 patients had second-line treatment failure with 4.07 per 100 person-year rate of failure. Being on anti-TB treatment [Rate ratio, RR = 2.57 (95% CI: 1.25–5.25)], not having optimal medication adherence [RR = 2.29 (95% CI: 1.09–4.78)], and not timely switched [RR = 5.89 (95% CI: 1.36–25.54)] were positively associated with treatment failure. Similarly, 44 (6.85%, 95% CI: 5–9%) of 642 patients died with 4.5 per 100 person-year rate of death. Being on advanced clinical condition [Sub distribution Hazard ratio, SHR = 2.49 (95% CI: 1.31–4.74)], not having optimal medication adherence [SHR = 2.65 (95% CI: 1.31–4.74)], lower CD4 cell counts, and high viral load measurement were positively associated with death.

**Conclusions:**

A significant number of patients had failed to respond to second-line therapy. A large number of patients had also died. Patient medical profile and monitoring practice were associated with treatment failure and death. Hence, patient-centered monitoring and interventions should be strengthened, besides treatment switch.

## Introduction

Globally, more than 26 million people living with HIV (PLWHIV) have accessed antiretroviral therapy (ART) by the end of 2020 [1], resulting in a significant reduction in HIV-related morbidity and mortality [2]. According to HIV estimates from the Ethiopian Public Health Institute (EPHI) and UNAIDS report, 60% of Ethiopia’s 737,186 HIV-positive people have received antiretroviral therapy in 2019 [3–5]. However, the number of HIV patients who switched to second-line antiretroviral therapy after failing first-line therapy is becoming a public health concern. A considerable number of patients worldwide had experienced treatment failure and switched to second-line antiretroviral therapy [6]. In Ethiopia, the proportion of people living with HIV (PLWHIV) on second-line antiretroviral therapy is also a rising issue; according to a systematic review, 15.9% (11.6–20.1%) of PLHIV had failed their first-line treatment failure, which requires switching to second-line therapy [7].

However, unlike first-line therapy, to date, very little is known about the outcomes of second-line antiretroviral therapy; rate of treatment failure, and death in Ethiopia context. Previously, few studies have been conducted in Ethiopia on treatment failure [8, 9]. However, those researches were done before the implementation of the viral load test (i.e., 2016) for monitoring and diagnosis of treatment failure [10, 11]. Viral load tests have a high sensitivity and positive predictive value in identifying treatment failure when compared to immunological and clinical failure diagnostic criteria [12, 13]. A patient having two consecutive viral load measurements greater than or equal to 1000 copies/ mL with three months of Enhanced Adherence Support (EAS) confirms the failure of the current treatment regimen [12, 13]. Factors that lead to poor treatment outcomes may relate to clinical and non-clinical determinants as well as vary from context to context.

HIV patients receiving second-line therapy are expected to achieve viral re-suppression as well as immunological and clinical improvement [8]. However, this may not be true for all patients. Poor second-line treatment outcomes have multidimensional consequences, at the individual, public, and policy levels. Individually, it increases the risk of drug resistance [14] and compromises the overall quality of life. At the public level, treatment failure raises the risk of HIV transmission and incurs high health care costs [15]. Second-line treatment failure has implications for how existing HIV programs are implemented in local settings. Furthermore, it jeopardizes the 95, 95, 95 fast track targets at the policy level [5, 16]. Hence, to prolong the use and efficacy of second-line therapy and to prevent further treatment failure assessing outcomes of patients on second-line antiretroviral therapy is so crucial. Therefore, this study was conducted to determine the rate of treatment failure and death and their predictors among PLWHIV on second-line antiretroviral therapy.

## Materials and methods

### Study design and setting

A retrospective cohort study was conducted at Dessie Comprehensive Specialized Hospital (DCSH) from October 2016 to November 2019. Dessie Comprehensive Specialized Hospital is located in the Amhara region, northeast Ethiopia, which is one of the top HIV burden areas in the nation [17]. Currently, 5557 and 1076 PLWHIV are receiving first-line antiretroviral therapy and ever enrolled in second-line therapy, respectively. During the three-year follow-up period, 686 adults PLWHIV started second-line antiretroviral therapy, of which, 642 patients had taken second-line antiretroviral therapy for more than six months. The viral load of the 642 PLWHIV was measured, and they were included in the study.

In Ethiopia, the current standard second-line regimen consists of a combination of three ARV drugs (at least two of which are new to the patient); two Nucleoside Reverse Transcriptase Inhibitors (NRTIs) and one Protease Inhibitor (PIs); Lopinavir/ritonavir (LPV/r) or Atazanavir /ritonavir (ATV/r) [12, 13]. Those PIs drugs are safer, more effective in viral suppression, and have a lower risk of resistance [18, 19].

Similarly, in Ethiopia, PLWHIV data are handled by SMART care, ART registration (logbooks) and chronic ART follow up form (patient chart). The patient chart is the main source of data, which is filled by a trained health professional and includes detailed data elements. The SMART care electronic database is another source of data for patients on ART. It is filled by trained non-health professionals/data clerks/ by reviewing patient charts. SMART care has only a few key data elements like CD4, TB screen, and viral load. ART registration (logbook) is similar to SMART care, except it is filled manually.

Antiretroviral treatment success is monitored using viral load (VL) measurement in which viral load measurement is done after initiation of ART at 6 months, 12 months, and every 12 months. In Ethiopia, viral load measurement was launched in 2016 on the top 20 burden areas including Dessie Comprehensive Specialized hospital [10, 11].

### Study population

Adult PLWHIV who have received second-line antiretroviral therapy at Dessie comprehensive specialized hospital is considered as the target population. Patients who were not taken second-line antiretroviral therapy for more than six months or those who had no viral load measurement after the commencement of second-line therapy were excluded from the analysis of treatment failure.

### Sample size

The minimum representative sample size was calculated using EPI-Info StatCalc by taking AHR: 1.68 for a factor of TB-preventive (INH) had not taken, 18.41% of outcome in the unexposed group in the study done in Ethiopia [9]. As well as, by considering other assumptions: 95% confidence level, 80% power, and one to one unexposed to exposed ratio, the final sample size were 404. However, all study participants who fulfilled the inclusion criteria and found in the study period were enrolled in the study (i.e., n = 642).

### Outcome variable and predictors

The primary outcome of this study was second-line treatment failure. A patient having two consecutive viral load measurements greater than or equal to 1000 copies/ mL with three months of Enhanced Adherence Support (EAS) confirms the failure of the current treatment regimen [12, 13]. Patients who are currently undergoing EAS intervention, on the other hand, are excluded from the treatment failure analysis. Because the patient’s fate was uncertain at the time of data collection, it will be decided once the EAS and the second viral load test are obtained.

Death was considered as a secondary outcome, which was confirmed by observing patient char and categorized as an event. Lost to follow-up is also defined as missing contact with the health care facility for more than three consecutive months. Patients who are alive on care or who have been transferred out, on the other hand, were considered censored.

#### Socio-demographic characteristics

Age, sex, marital status, educational status, and disclosure status.

#### Patient medical profiles

Year on ART, body mass index (BMI), functional status, WHO clinical stage, TB treatment status, TB-preventive therapy (INH), CD4 cell/mm^3^, viral load, first-line ART regimen before the switch, second-line ART regimen, medication adherence, and time between first enrolled to EAS and initiation of second-line therapy. Patients were considered timely switched if they shifted to second-line therapy within 3–6 months of enrollment in EAS [12, 13].

Medication adherence was assessed by reviewing the patient follow-up form, which was collected via self-report and filled by a health professional. Patients who are taking > = 95% of the prescribed medication are considered as optimal adherence unless a patient is categorized as not optimal adherence. This cutoff point was adopted following national and WHO anti-retroviral guideline recommendations [12, 13].

### Data collection procedures

The data were collected using a standardized data extraction checklist by reviewing chronic HIV follow-up form (patient chart or card), ART registration books, and SMART care electronic database. The extraction was made by reviewing chronic HIV follow-up form (patient chart or card), ART registration book, and SMART care electronic database for patients who started second-line antiretroviral therapy from October 2016 to November 2019. The extraction sheet was prepared in accordance with the national consolidated antiretroviral guideline [13]. A patient chart or card was retrieved using the patient Medical Record Number (MRN) and unique ART registration number. The qualities of data were secured by triangulation of the above data sources; minimizing data incompleteness and inconsistency, using a pre-tested extraction checklist, employing trained data collectors, and conducting on-site supervision.

### Statistical analysis

Data were cleaned and entered into EpiData Version 3.1 software. Then, exported to Stata version 14 for further analysis. For categorical variables, frequency (%) was utilized, while for skewed distributed continuous variables, median (interquartile range, IQR) were employed after checking the normality assumption using Kolmogorov—Smirnov and Shapiro-Wilk test. The incidence rate of treatment failure and death was calculated using person-time of observations. Person-time is the sum of the number of years contributed by study participants in the follow-up period.

A Poisson regression model was computed to explore predictors of treatment failure. The number of treatment failures during three year follow-up period is a rare count outcome, hence Poisson regression was computed. Poisson distribution assumption was checked via Kolmogorov-Smirnov test and it was non-significant (P-value > 0.05), which is a Poisson assumption not violated. Similarly, to identify predictors of death, the Competitive Risk Survival Model (CRS-M) was computed by considering patients who lost from care as competitive event. The loss could be caused by clinical deterioration or death that is not reported to the health facility. In Ethiopia, HIV-related death is more common, particularly among patients who have failed first-line treatment and lost from care. According to the UNAIDS-2019 HIV report, 11,000 Ethiopians die each year as a result of HIV/AIDS [20]. In variable screening, first bi-variable analysis was done for each explanatory variable, and variables having P-value less than 0.25 were imported to the multivariable model. In the multivariable Poisson and Competitive Risk Survival Model, variables having P-value <0.05 were considered as statistically significant and the effect size was presented using 95% CI.

### Ethics approval and consent to participate

This study was conducted in accordance with the declaration of Helsinki. Ethical approval was secured from the Institutional Review Board (IRB) of Bahir Dar University, College of Medicine and Health Science with reference number 00224/2020. Further, authorization was obtained from Dessie Comprehensive Specialized Hospital to use anonymized data. We ensure that all data/samples were fully anonymized before accessing them. The IRB of Bahir Dar University’s College of Medicine and Health Science waived the requirement for informed consent because we used document review as a data source. Besides, the confidentiality of obtained information assurance is made by using code numbers rather than personal identifiers and by keeping the checklist locked.

## Results

### Description of study participants

**Fig 1:**
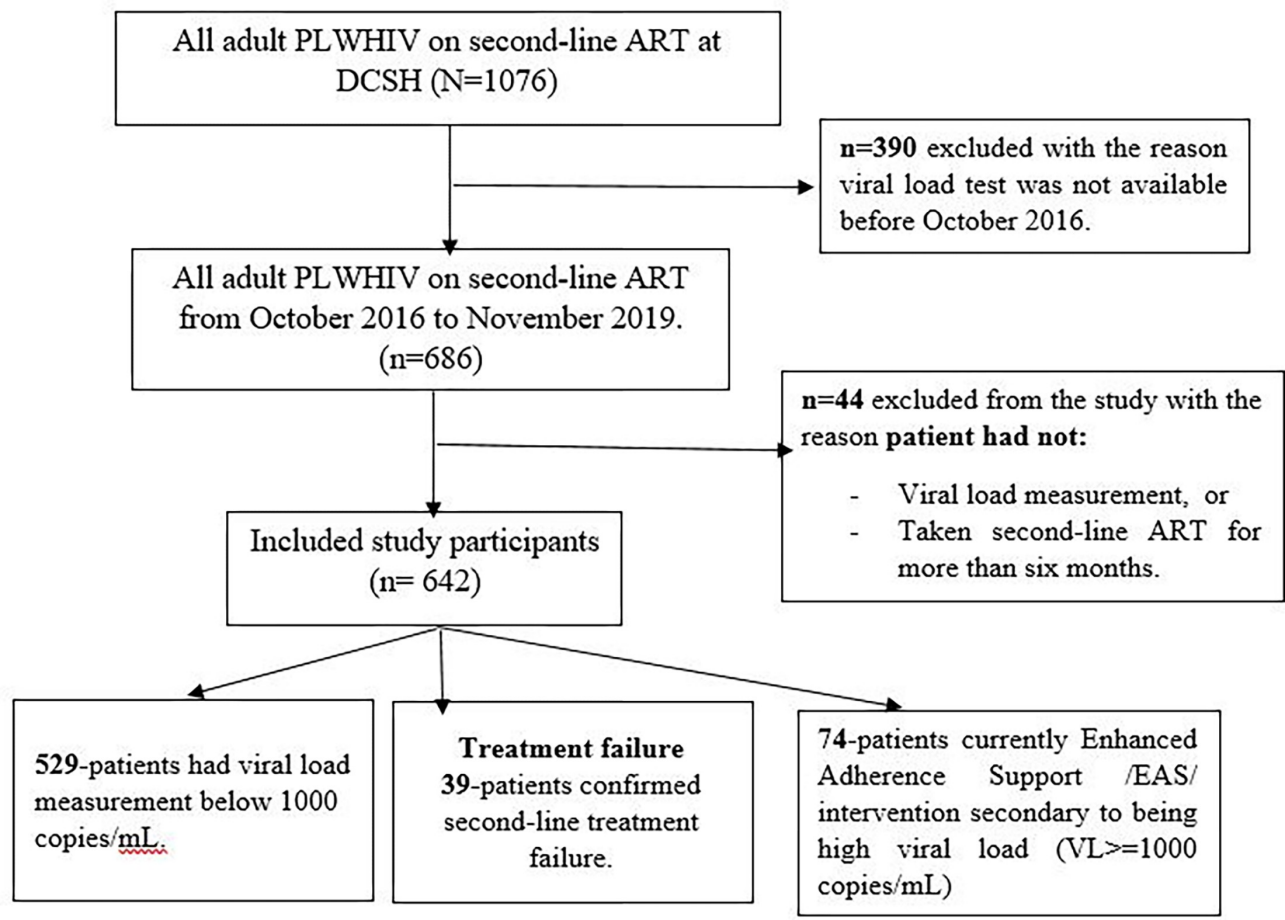
Second-line treatment failure among PLWHIV on second-line therapy at Dessie Comprehensive Specialized Hospital/DCSH/, northeast Ethiopia, from October 2016 to November 2019. (n = 642).

Out of 642 PLWHIV who were receiving second-line antiretroviral therapy, 529(82.39%) had viral load measurement below 1000 copies/mL, 74 were currently on EAS intervention and 39 patients had confirmed second-line treatment failure Fig 1.

Regarding socio-demographic profiles, 359 (55.9%) and 298(46.4%) of the 642 participants were female and married, respectively. One-third of the participants had not attended formal education, and 181(28.2%) were unemployed. Participants’ median (IQR) age and year on ART were 35(27–42) and 8(5.4–10.1) years, respectively (Table 1).

**Table 1:** Socio-demographic characteristics of PLWHIV on second-line antiretroviral therapy at Dessie Comprehensive Specialized Hospital, northeast Ethiopia, October 2016—November 2019. (n = 642).

Characteristics	Total (642) N (%)	Treatment failure(39) N (%)	Death (44) N (%)
Sex			
Female	359(55.9)	17(43.59)	22(50.00)
Male	263(44.1)	22(56.41)	22(50.00)
Marital status			
Single	199(31.0)	16(41.03)	12(27.27)
Married	298(46.4)	9(23.08)	12(27.27)
Divorce	83(12.9)	9(23.08)	11(25.00)
Widowed	62(9.7)	5(11.82)	9(20.50)
Religion			
Orthodox	282(43.9)	18(46.15)	15(34.10)
Muslim	325(50.6)	19(48.72)	27(61.40)
Protestant	23(3.6)	1(2.56)	2(4.50)
Catholic	12(1.90)	1(2.56)	0(0.00)
Educational level			
Not formally educated	216(33.6)	18(46.15)	19(43.18)
Primary	182(28.3)	8(20.51)	13(29.55)
Secondary	157(24.5)	7(17.95)	7(15.90)
Tertiary	87(13.6)	6(15.38)	5(11.36)
Occupational status			
Government employee	65(10.10)	4(10.26)	4(9.09)
Housewife	145(22.6)	9(23.08)	11(25.00)
Merchant	103(16.0)	6(15.38)	8(18.18)
Farmer	114(17.8)	4(10.26)	8(18.18)
Unemployed	181(28.2)	15(38.46)	13(29.55)
Private employee	34(5.3)	1(2.56)	0(0.00)
Disclosure status			
Yes	538(83.8)	30(76.92)	36(81.82)
No	104(16.2)	9(23.08)	8(18.18)
Duration on antiretroviral therapy, median (IQR) (year)	8(5.4–10.1)	8(7–11)	7(4.5–11)
Age, median (IQR)(year)	35(27–42)	35(25–45)	35(26–45)

### Medical profile of PLWHIV

Out of 642 participants, about 466(72.8%) patients had BMI > = 18.5 kg/m^2^ and 140(21.8%) had CD4 cell counts greater than 450 cells/mm^3^. Similarly, 85(13.2%) and 19(3%) patients were in advanced clinical stages and bedridden. Fifteen percent of PLWHIV were on anti-TB treatment and 204(31.8%) had not taken TB preventive therapy (INH). TDF-3TC-EFV; 219(34.1%) and TDF-3TC-ATV/r (283(44.1%) were the most prescribed first and second-line antiretroviral therapy.

Regarding medication adherence and drug substitution, 574(89.4%) patients had optimal second-line medication adherence while 448(69.8%) patients had no drug substitution history. The median time between first enrollments in EAS to initiation of second-line therapy was 5(IQR 3–8) months and 431(67.13%) participants were delayed to switch in a timely. Regarding viral load measurement, 113(17.6%) out of 642 patients had a high viral load measurement (VL> = 1000 copies/ml after PIs exposure (Table 2).

**Table 2:** Clinical characteristics of PLWHIV on second-line antiretroviral therapy, at Dessie Comprehensive Specialized Hospital, northeast Ethiopia, October 2016—November 2019.

Patients medical profiles	Total (642) N (%)	Treatment failure (39), N (%)	Death (44) N (%)
BMI: > = 18.5 kg/m^2^	466(72.6)	18(46.15)	14(31.82)
16–18.4 kg/m^2^	113(17.6)	14(35.90)	14(31.82)
< 16 kg/m^2^	63(9.8)	7(17.95)	16(36.36)
Functional status			
Workable	542(84.4)	26(66.67)	20(45.45)
Ambulatory	81(12.6)	11(28.21)	18(40.90)
Bedridden	19(3.0)	2(5.13)	16(13.65)
WHO clinical stages: I and II	557(86.7)	28(71.79)	20(45.45)
III and IV	85(13.3)	11(28.21)	24(54.55)
TB treatment status			
On anti-TB treatment	93(14.5)	15(38.46)	21(47.73)
Not on TB treatment	549(85.5)	24(61.54)	23(52.27)
TB-Prophylaxis(INH): Had not taken	204(31.8)	29(74.36)	37(84.09)
CD4 cells/mm^3^: < = 450 cells/mm^3^.	502(78.2)	34(87.18)	21(47.73)
> 450 cells/mm^3^.	140(47.2)	5(12.82)	23(52.27)
First-line ARV regimen			
AZT-3TC-NVP	195(30.4)	15(38.46)	15(34.09)
AZT-3TC-EFV	144(22.4)	4(10.26)	5(11.36)
TDF-3TC-EFV	219(34.1)	16(41.03)	17(38.64)
TDF-3TC-NVP	84(13.1)	4(10.26)	7(15.90)
First-line drug substitution history: Yes	194(30.2)	17(43.59)	17(38.64)
Second-line ARV regimen			
AZT-3TC-LPV/r	17(2.6)	1(2.56)	1(2.27)
AZT-3TC-ATV/r	238(37.1)	14(35.90)	24(54.54)
TDF-3TC-LPV/r	21(3.3)	2(5.13)	12(27.27)
TDF-3TC-ATV/r	283(44.1)	18(46.15)	1(2.27)
ABC-3TC-LPV/r	13(2.0)	1(2.56)	2(4.54)
ABC-3TC-ATV/r	70(10.9)	3(7.69)	4(9.09)
Medication Adherence: Optimal adherence	574(89.4)	27(69.23)	22(50.00)
Not optimal adherence	68(10.6)	12(30.77)	22(50.00)
Weight, median (IQR) (kg)	50(44–57)	48(43–52)	43(40–49)
HIV viral load at therapy switch, median (IQR) (copies/mL)	13195	26191	42890
	(4140–52753)	(9120–86372)	(13066–98400)
The time between first enrollment to EAS and initiation of second-line therapy. median (IQR) month	5(3–8)	7(5–9)	7(5–9)
Delayed to switch	431(67.13)	37(94.9)	37(84.1)
Timely switch	211(32.87)	2(5.1)	7(15.9)
Last HIV viral load result after PIs exposure			
High viral load (VL> = 1000 copies/mL)	529(82.39)		14(31.82)
Not high viral load	113(17.61)		30(68.18)

### Rate of second-line treatment failure and predictors

By excluding patients currently on EAS intervention, 39 (6.87%, 95%CI: 5–9.2%) of 568 patients had second-line treatment failure in 959.3 years of follow-up. The incidence rate of treatment failure was 4.07 per 100 person-year, which can be found by dividing the total number of treatment failure cases by follow-up year (i.e., 39/959.3 = 4.07 per 100 person-year).

Patients who were delayed to switch timely were 5.89 times more at risk of second-line treatment failure as compared with timely switched, while holding all the other variables in the model remain constant [Adjusted Rate Ratio, ARR = 5.89 (95% CI:1.36–25.54)]. Patients who were on anti-TB treatment were 2.57 times more prone to second-line treatment failure as compared with counterparts [ARR = 2.57 (95% CI: 1.25–5.25)]. Similarly, patients who had not optimal medication adherence were 2.29 times more at risk of second-line treatment failure as compared with counterparts [ARR = 2.29 (95% CI: 1.09–4.78)] (Table 3).

**Table 3:** Bi-variable and multivariable poisson regression model on determinants of second-line treatment failure among PLWHIV on second-line therapy, Dessie comprehensive Specialized Hospital, northeast Ethiopia, October 2016—November 2019. (n = 568).

Covariate	Bi-variable	Multivariable	
	CRR(95% CI)	ARR(95% CI)	P-value
Year on ART	1.07(0.96–1.19)		
Drug substitution history: no	0.53(0.28–0.99)		
Time to switch: delayed to switch timely	10.61(2.55–44.01)	5.89(1.36–25.54).	0.018
HIV viral load at therapy switch (ref: 1000–4140 copies/mL)			
4141–13195 copies/mL	3.51(0.96–12.75)		
13196–52753 copies/mL	4.05(1.11–14.72)		
52756–702127 copies/mL	6.48(1.88–22.24)		
CD4 cell counts: > 450 cells/m^3^	0.49(0.19–1.24)		
WHO clinical stages: Stage IV and III	3.04(1.51–6.10)		
Being on anti -TB treatment	4.67(2.45–8.90)	2.57(1.25–5.25)	0.01
INH status: Had not taken INH	2.44(1.19–5.02)		
Medication adherence: not optimal medication adherence (< 95%)	5.43(2.75–10.71)	2.29(1.09–4.78).	0.028

### Death rate and predictors

In this study, 44(6.85%, 95CI: 5–9%) of 642 patients died with 4.5 per 100 person-year rates of death in 968.3 year observation period. The death rate was found by dividing the total number of deaths by the total follow-up period (i.e. 44/968.3 = 4.5 per 100 person). Out of total deaths, 23(52.2%) and 15(34%) deaths were reported at years one and two, respectively (Table 4). During three years follow-up period, 19 (3%), 70(10.9%), and 509(79.3%) of 642 patients were lost to follow-up, transferred out and alive on care, respectively.

**Table 4:** Actuarial table for death among PLWHIV on second-line therapy, Dessie comprehensive specialized Hospital, northeast Ethiopia, October 2016—November 2019. (n = 642).

Time interval in month	Total	Deaths	Censored	Cumulative failure(Death)	95% CI
0–12	642	23	184	0.0418	0.028–0.0623
12–24	435	15	211	0.0854	0.0623–0.1167
24–36	209	5	163	0.1213	0.0868–0.1682
36–48	41	1	40	0.1631	0.0935–0.2760
Total		44	598		

### Competitive risk survival model

A competitive risk survival model showed that at therapy switch, patients who had an advanced clinical condition and lower CD4 cell counts were more at risk of death. After initiation of second-line therapy, patients who had not optimal medication adherence and viral load greater than or equal to 1000 copies/mL were more at risk of death.

Patients who had advanced clinical conditions were 2.49 times more at risk of death as compared with the counterpart [Sub distribution Hazard ratio, SHR = 2.49 (95% CI: 1.31–4.74)]. Similarly, for every CD4 cell count increment, on average death rate decreased by 0.4%, while holding all other variables in the model remained constant [SHR = 0.996(95% CI: (0.994–0.999)].

Patients who had not optimal medication adherence were 2.65 times more at risk of death as compared with counterparts [SHR = 2.65, (95% CI: 1.29–5.43)]. Similarly, PLWHIV who had a viral load greater than or equal to 1000 copies/mL were seven times more at risk of death as compared with counterparts, while holding all other variables in the model constant [SHR = 6.97 (95% CI: 3.3–14.74)] (Table 5).

**Table 5:** Bi-variable and multivariable competitive risk model on determinants of death among PLWHIV on second-line therapy, Dessie Comprehensive Specialized Hospital, northeast Ethiopia, October 2016—November 19. (n = 642).

Covariates	Bi-variable	Multivariable	
	CSHR(95% CI)	ASHR(95% CI)	P-value
Age	1.01(0.97–1.03)		
Sex: Male	1.28(0.71–2.3)		
Educational status: not formally educated	1.56(0.86–2.82)		
year on ART	0.96(0.86–1.08)		
BMI <18.5 kg/m^2^	5.83(3.10–10.97)		
HIV viral load at therapy switch (copies/mL)	1.02(1.00–1.04)		
CD4 cell /m^3^	0.995(0.92–0.97)	0.996(0.994–0.999)	0.009
WHO clinical stages: IV & III	8.54(4.78–15.28)	2.49(1.31–4.74)	0.005
INH status: Had not taken INH	4.16(1.85–9.32)		
Medication adherence: not optimal medication adherence (< 95%)	10.22(5.72–18.25)	2.65(1.29–5.43)	0.008
Last HIV viral load result after PIs exposure: High HIV viral load (VL > = 1000 copies/mL)	14.49(7.85–24.75)	6.97(3.3–14.74)	<0.001

SHR: Sub distribution Hazard ratio

## Discussion

Seven percent of PLWHIV had experienced second-line treatment failure during three follow-up periods. This finding is lower than studies conducted in the northwest and northern Ethiopia; 18.8% and 25.11%, respectively [6, 17]. Similarly, the incidence of second-line treatment failure is lower than other study findings [8, 9, 21, 22]. The introduction of viral load tests in Ethiopia allows for early identification of treatment failure ahead of immunological and clinical deterioration. Consequently, patients will switch before clinical worsen, resulting in having a better treatment response for the new regimen and a decrease in the likelihood of treatment failure. Similarly, after initiation of second-line therapy, viral load monitoring allows in screening and enrolled high viral load patients into enhanced adherence support. A systematic review showed that 70% high viral load patients will re-suppress following adherence intervention [23].

Generally, this finding showed that a significant number of patients had experienced second-line treatment failure. This has both clinical and public health implications. Clinically, it increases the risk of drug resistance and the demand for high-cost third-line antiretroviral therapy. At the public level, it also increases the chance of HIV transmission, even resistant strain. Hence, due emphasis needs for HIV patients receiving second-line therapy, besides treatment switch.

Being on anti-TB treatment and second-line treatment failure was positively associated, which is supported by studies conducted in Ethiopia and Uganda [21, 24]. In line with this, studies showed that having an advanced clinical condition at treatment switch is a risk for treatment failure [8, 9, 21, 22] and adverse drug reaction [25]. This association is possibly explained by TB-HIV co-infected patients will have more pill burden and drug side effects, resulting in poor medication adherence and treatment failure. Furthermore, there might be an interaction of TB treatment drugs and second-line antiretroviral therapy; rifampicin, a potent cytochrome P450 3A4 liver enzyme inducer, which significantly reduces the serum levels of the PIs drugs and reduce the rate of viral re-suppression. According to WHO—2016 and national consolidate ART guideline, for TB-HIV Co-infected patients the dose of lopinavir/r should be adjusted; doubling the daily dose (i.e. LPV/r 800 mg/200 mg twice daily) or super-boosted dose of RTV (i.e. LPV/r 400 mg/400 mg twice daily) [12, 13]. However, this may also lead to drug intolerance and poor medication adherence.

A delay in switching after treatment failure was associated with second-line treatment failure. In line with this, studies done in Africa showed that delay to switch after first-line treatment failure leads to poor treatment outcomes [26–28]. This treatment gap will give sufficient time for viral replication, development of resistance strain, which results in poor treatment progress. Failure to switch timely manner may relate to the patient, health care provider, or health facility factors, which may require further investigation. Non-optimal medication adherence was directly associated with second-line treatment failure, which is supported by many studies [9, 21, 22, 29–31]. As shown in many studies, poor medication adherence is a primary cause of first-line treatment failure. Switching such patients without solving adherence barriers might be led to further treatment failure.

Regarding the death of PLWHIV, seven percent of participants were died, of which more than fifty percent were reported with in the first year after the starting second-line therapy. This finding is lower than other studies, including a study done in Ethiopia [31–33]. This disagreement may also relate to diagnosis criteria of treatment failure. In this study, patients enrolled in a second-line program using virological criteria. This might put patients in better clinical condition, treatment compliance, and treatment outcomes as compared with patients who switched via immunological or clinical criteria.

Having an advanced clinical condition and lower CD4 cell count at therapy switch was significantly associated with death, which is consistent with studies done in other settings [32, 34–37]. This finding implies that having such clinical profiles at therapy switch is an early indicator for the fate of subsequent treatment outcomes. Hence, such patients require systematic identification and close monitoring to avert unwanted treatment outcomes.

Further, patients having poor medication adherence and high viral load (i.e, > = 1000 copies/mL) were also positively associated with the risk of death. This finding is in agreement with other studies [31–35]. Hence, different strategies should be crafted and implemented to enhance medication adherence and viral re-suppression like; implementation of enhanced adherence counseling for the first six months of second-line therapy, monitoring of patients by the same clinician in repeated visits as well as timely viral load monitoring is highly recommended.

### Strengths of the study

First, the study is the first of its kind in Ethiopia to provide first-hand information to improve the outcomes of patients on second-line therapy after the implementation of the viral load test. Second, it was conducted on a large sample size as well as used different data sources (Chronic HIV follow up form, ART registration book, and SMART care electronic database) that increases consistency as well as minimize incompleteness.

### External validity

The finding of this research can be also extrapolated in other low-income countries’ settings where they implement similar WHO treatment modalities, outcome ascertainment, and have a similar socio-demographic contexts [38].

### Limitation of the study

This study failed to collect and analyze data on behavioral, social, and psychological factors as well as facility-level determinants of treatment failure and death. Those limitations were related to the retrospective cohort design, in which data were collected by reviewing medical charts Furthermore, this study was unable to get and consider other clinical profiles such as organ function results, drug resistance tests. Therefore, confounding through the unmeasured covariates need to be considered while interpreting the reported associations. Another limitation of this study was fail to report the specific cause of death.

## Conclusions

A large percentage of patients had not responded to second-line treatment. A substantial proportion of the patients had also died. Treatment failure and death were linked to the patient’s medical profile and monitoring practices. As a result, in addition to treatment switching, patient-centered monitoring and treatments should be reinforced.

## Supporting information

S1 File (DOCX)

## List of abbreviations

ART: antiretroviral therapy
PLHIV: People Living with HIV
VL: viral load
NRTIs: Nucleoside Reverse Transcriptase Inhibitors (NRTIs)
NNRTIs: Non-Nucleoside Reverse Transcriptase Inhibitors (NNRTIs)
INSTIs: Integrase strand transfer Inhibitors
PIs: Protease Inhibitors
DTG: Dolutegravir 3TC: Lamivudine
ABC: Abacavir
AZT: Zidovudine
TDF: Tenofovir
EFV: Efavirenz
NVP: Nevirapine
LPV/r: Lopinavir/ ritonavir
ATV/r: Atazanavir /ritonavir
BMI: Body Mass Index
IQR: Interquartile range
ADR: adverse drug reaction
INH: Isoniazid preventive therapy
MRI: Medical Record Number
DHIS2: District health information system
RR: Rate Ratio
SHR: Sub Distribution Hazard Ratio
