# Supplementary material for: Treatment failure, death, and predictors among PLWHIV on second-line antiretroviral therapy in Dessie Comprehensive Specialized Hospital, northeast Ethiopia: A retrospective cohort study
Source: PLoS One. 2022 Jun 1;17(6):e0269235. doi: 10.1371/journal.pone.0269235 (PMC9159605; doi:10.1371/journal.pone.0269235)

**Data Extraction Sheet on Treatment failure, death, and predictors among PLWHIV on Second-Line Antiretroviral therapy at Dessie Comprehensive Specialized Hospital, northeast Ethiopia: A retrospective cohort study**

**.**

| S. No | Age | Sex (M or F) | Marital status | Religion | Educational level | Occupation status | Does anyone else know about your HIV Status/ 1.Yes, 2. No/ |
| --- | --- | --- | --- | --- | --- | --- | --- |
|  |  |  |  |  |  |  |  |
|  |  |  |  |  |  |  |  |
|  |  |  |  |  |  |  |  |
|  |  |  |  |  |  |  |  |
|  |  |  |  |  |  |  |  |
|  |  |  |  |  |  |  |  |
|  |  |  |  |  |  |  |  |
|  |  |  |  |  |  |  |  |

1. **Socio-demographic profiles *(Data sources: Intake A, B, and Chronic follow up form)***


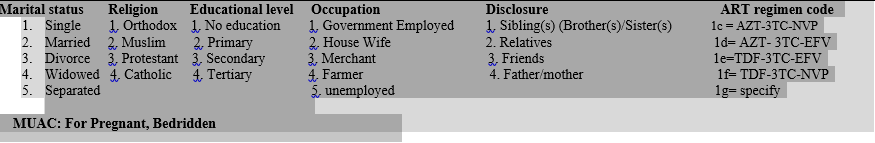


1. **Clinical profiles** *(Chronic follow up form and ART registration)*

| **S. No** | **Months on ART** since the first ART initiation | **Date of 2^nd^ line initiated** | Weight (Kg) | Height (m) | **BMI** or MUAC | Functional Status(W,A,B) | **WHO** clinical stages(T-stage) | **TB** status | **INH** status | **OI** (HIV related cancers) | CD4 cells /mm3 | Viral load copies/mL | Hemoglobin (Hgb.) | **CPT** (0. on CPT, 1. Not) | **FPT**(0. on FPT, 1. not on FPT) | **Other medications** (1. yes 2. no) | Reported **adherenc**e status | **1^st^- line ARV regimen** | Drug **substitution history** while in First line therapy /1.yes, 2. no/ | **2^nd^ line ARV regimen** | Reported drug **Side effect** in **the first six** months | Duration b/n 1^st^ VF & Start of 2^nd^ line ART |
| --- | --- | --- | --- | --- | --- | --- | --- | --- | --- | --- | --- | --- | --- | --- | --- | --- | --- | --- | --- | --- | --- | --- |
|  |  |  |  |  |  |  |  |  |  |  |  |  |  |  |  |  |  |  |  |  |  |  |
|  |  |  |  |  |  |  |  |  |  |  |  |  |  |  |  |  |  |  |  |  |  |  |
|  |  |  |  |  |  |  |  |  |  |  |  |  |  |  |  |  |  |  |  |  |  |  |
|  |  |  |  |  |  |  |  |  |  |  |  |  |  |  |  |  |  |  |  |  |  |  |
|  |  |  |  |  |  |  |  |  |  |  |  |  |  |  |  |  |  |  |  |  |  |  |
|  |  |  |  |  |  |  |  |  |  |  |  |  |  |  |  |  |  |  |  |  |  |  |
|  |  |  |  |  |  |  |  |  |  |  |  |  |  |  |  |  |  |  |  |  |  |  |


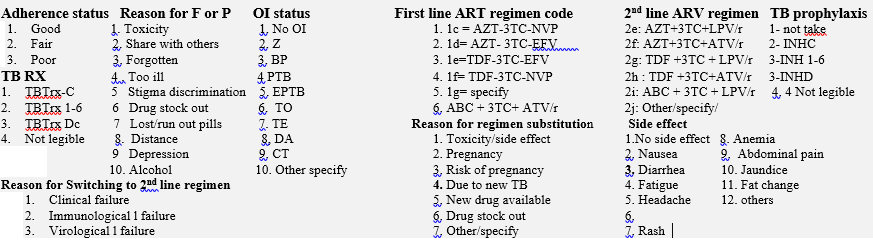


1. **Outcomes Measurements** *(Chronic follow up form and ART registration)*

| **S. No** | **Viral load Measurements** | | | | | |  | **Death status** | |  |
| --- | --- | --- | --- | --- | --- | --- | --- | --- | --- | --- |
|  | 6-month | 12- month | 24-Month | 36-Month | Treatment failure status (yes or not ) | Last date of visit HF |  | Status | Last date of visit HF or reported date of death or LF |  |
|  |  |  |  |  |  |  |  |  |  |  |
|  |  |  |  |  |  |  |  |  |  |  |
|  |  |  |  |  |  |  |  |  |  |  |
|  |  |  |  |  |  |  |  |  |  |  |
|  |  |  |  |  |  |  |  |  |  |  |
|  |  |  |  |  |  |  |  |  |  |  |
|  |  |  |  |  |  |  |  |  |  |  |
|  |  |  |  |  |  |  |  |  |  |  |
|  |  |  |  |  |  |  |  |  |  |  |


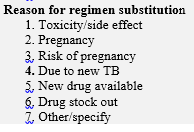

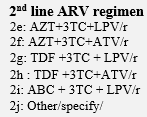

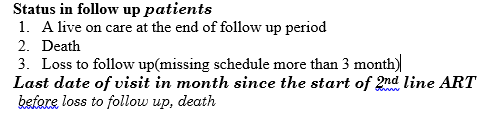


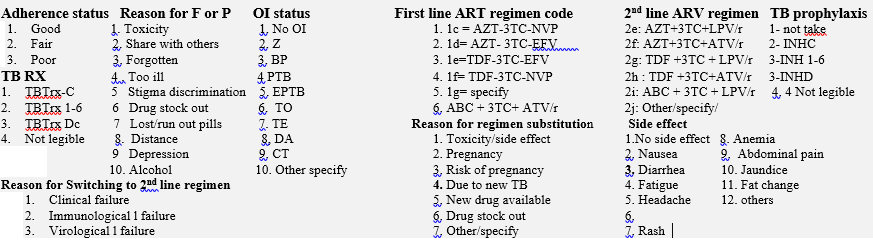

Supplement: S1 File — (DOCX) [file pone.0269235.s001.docx]
